# The chiasmata systems of Scottish *Chysolinalatecincta* (Demaison, 1896) (Coleoptera, Chrysomelidae)

**DOI:** 10.3897/CompCytogen.v15.i4.68309

**Published:** 2021-09-28

**Authors:** Eduard Petitpierre

**Affiliations:** 1 Dept. of Biology, University of Balearic Islands, 07122, Palma de Mallorca, Spain University of Balearic Islands Palma de Mallorca Spain

**Keywords:** Coleoptera, chiasmata, Chrysomelidae, *Chrysolinalatecinctaintermedia*, meiosis

## Abstract

The meiotic systems of some Scottish individuals of the rare Chrysolinalatecinctassp.intermedia (Franz, 1938) have been analyzed from meiotic cells at diakinesis to study the types of chromosomal bivalents and the number and locations of their chiasmata. The mean number of unichiasmate was about two-thirds and that of bichiasmate bivalents about one-third. Most chiasmata were at distal positions and there were no pairwise statistically significant differences in the mean number of chiasmata and those of unichiasmate and bichiasmate bivalents between the three surveyed geographic sources of these Scottish individuals. However, pairwise significant differences were found in the mean number of proximal + interstitial chiasmata between Loch Etive (Argyllshire) and both Orkney and Shetland Islands individuals. The presumed higher values of genetic recombination due to the proximal + interstitial chiasmata with regard to the prevailing distal ones, might provide a slight selective advantage to the insular individuals against the more extreme climates of both islands compared with the Loch Etive site.

## Introduction

*Chrysolinalatecincta* (Demaison, 1896) is a species distributed in western and central Europe from southern Norway to Spain and from Scotland to the Alps and Apennines (Kippenberg 2010). There are ten described subspecies that include the rare Ch.l.ssp.intermedia (Franz, 1938), which is found in a few cliff Scottish localities (Hubble 2017; Bienkowski 2019). A cytogenetic analysis of three male individuals of Ch.latecinctassp.latecincta from southern France has shown 2n = 24 chromosomes, with four large and seven small autosome pairs, plus a medium size X and a very small Y sex-chromosomes (Petitpierre 2000). In agreement with this size asymmetric karyotype the meiotic metaphases I display a male meioformula of 11 + Xy_p_, with four large and seven small autosome bivalents and the achiasmate “parachute-like” Xy_p_ sex-chromosome (Petitpierre 1999). The aim of this paper is to get an insight on the chiasmate systems of the Scottish Ch.l.ssp.intermedia from three distinct geographic sources to compare them with data previously published from the above French typical *Ch.latecincta* subspecies and with further others closely related species in the same subgenus Stichoptera (Motschulsky, 1860). Moreover, the possible differences in chiasmate systems between these Scottish individuals will be also studied.

## Materials and methods

One male individual from Orkney Islands, another one from Shetland Islands and two from Loch Etive, Argyllshire, in mainland Scotland, were chromosomally surveyed. The four samples of preserved testes in ethanol: glacial acetic acid (3:1) were sent to our laboratory at the University of Balearic Islands, in Palma de Mallorca, for their chromosomal analyses. We used a simple technique reported by Petitpierre et al. (1998) to obtain slides that were later stained in 4% diluted Giemsa in tap water before their final checking under a ZEISS AXIOSKOP microscope.

## Results

The observations and scoring of chiasmata were performed on cells at meiotic diakinesis in order to know the number of unichiasmate and bichiasmate bivalents and their locations as proximal, interstitial or distal, in each type of meiotic configurations, according with the drawings by John and Lewis (1975, p. 50). Results are given in Table 1. From the total number of our 1264 scored bivalents, 68.2% were unichiasmate, mostly rod-shaped and much less often cross-shaped, and 31.8% bichiasmate, mostly ring-shaped. The percentage of distal chiasmata was clearly exceeding, from 87.7% to 96.3%, to those of proximal + interstitial ones. The mean total number of chiasmata per cell was very similar in all individuals because the four large autosome pairs were responsible for at least two and mostly three or four bichiasmate bivalents in all cells scored. The analysis of statistically significant differences between individuals from the three sites (Table 2, *t*-Student test), showed that the number of proximal + interstitial chiasmata varied significantly (**P < 0.01) between the Loch Etive individuals and those of Shetland and Orkney Islands individuals; a less significantly difference was found (*P < 0.05) in the mean number of distal chiasmata between Loch Etive and Orkney Islands individuals.

**Table 1.** Number of each class of chiasma and mean values per cell in Scottish individuals of Chrysolinalatecinctassp.intermedia. pro. = proximal, int. = interstitial, nr. = number.

**Islands**	**Loch Etive**	**Orkney Islands**	**Shetland Islands**
Cells scored	75	11	29
bivalents scored	825	121	319
bichiasmate bivalents	254 (30.8%)	42 (34.7%)	106 (33.2%)
unichiasmate bivalents	570 (69.2%)	79 (65.3%)	213 (66.8%)
total nr. of chiasmata	1078	163	425
mean nr. of chiasmata	14.373±0.117	14.818±0.157	14.60±0.162
nr. of distal chiasmata	1038 (96.3%)	143 (87.7%)	392 (92.2%)
mean nr. of distal chiasmata	x = 13.84±0.122	x = 13.00±0.374	x = 13.517±0.297
pro. + int. nr. of chiasmata	40 (3.7%)	20 (12.3%)	33 (7.76%)
mean pro.+ int. nr. of chiasmata	x = 0.533±0.089	x=1.818±0.395	x = 1.138±0.207

**Table 2.** Pairwise comparisons for chiasma mean numbers between individuals from the three Scottish sites of Chrysolinalatecinctassp.intermedia. pro. = proximal, int. = interstitial.

**Islands**	**Bichiasmate**	**Unichiasmate**	**Pro. + Int.**	**Distal**	**Total**
Loch Etive & Orkney Isl.	0.872	1.398	**3.000 *2.228	*2.228	1.422
Loch Etive & Shetland Isl.	0.845	1.321	**3.563 1.022	1.022	1.433
Orkney Islands & Shetland Isl.	0.370	0.506	1.453 1.122	1.022	0.492

**P < 0.01 *P < 0.05

## Discussion and conclusions

From the total number of our 1265 studied bivalents in *Ch.latecinctaintermedia* 68.2% were unichiasmate, mostly rod-shaped, and 31.8% bichiasmate, mostly ring-shaped (Table 1).

The mean number of chiasmata per cell in Scottish *Ch.latecincta* were very similar in all studied individuals from the three sites, 14.4 to 14.8, due to the regular formation of a single chiasma in the seven smaller pairs of autosomes, and a varying number of one or two chiasmata in each of the four larger pairs (Table 1). The small differences in mean total number of chiasmata and in both bichiasmate and unichiasmate bivalent numbers between Scottish individuals were not statistically significant (Table 2). These results are in agreement with those obtained in other *Ch.latecincta* individuals from Southern France, whose four larger autosomes correspond to 57.44% of the total complement length and are responsible for all the bichiasmate bivalents scored (Petitpierre 2000). Another closely related species, *Ch.oceanoripensis* Bourdonné, Doguet & Petitpierre, 2013 (*Ch.ripoceanensis*; Petitpierre 2000), from Southwestern France, and belonging to the same subgenus Stichoptera Motschulsky, 1860, has 2n = 24 (Xy_p_) too, and displays equally 14 chiasmata per cell, but a bit lower average of 27.3% of bichiasmate bivalents than those shown in Scottish individuals, from 30.8% to 34.7%. The finding of a clear prevalence of distal chiasmata in Scottish individuals (Table 1), agrees with the results found in other species of the subgenus Stichoptera, *Ch.oceanoripensis* and *Ch.gypsophilae* (Petitpierre 2000). A similar distal localization has been reported in *Pleocoma* Le Conte, 1856 (Scarabaeidae), *Pissodes* Germar, 1817 (Curculionidae) and even in almost all the New World Oedionychina subtribe of chrysomelids (Smith and Virkki 1978). In two hispini Chrysomelidae the same prevalence of distal localisations of chiasmata was found, namely, more than 85% of them in *Dicladispatestacea* (Linnaeus, 1767) and 75% in *Polyconiacaroli* (Leprieur, 1883), were distally positioned (Alegre and Petitpierre 1990).

The significant difference in the mean number of proximal + interstitial chiasmata between Loch Etive and both Shetland and Orkney Islands (Table 2), might be related to environmental differences between these sites. The recombination level derived from these proximal + interstitial chiasmata is presumably higher than that derived from distal chiasmata, due to the much larger size of chromatids exchanged. However, it should be noted that although extreme environments, such as low temperatures, may have some influence on chiasma formation, their effects on chiasma frequency are ambiguous (Wilson 1959). The 7.1 °C mean year temperature in Shetland Islands is lower than that of 7.8 °C in Orkney Islands, and both are lower to that of 8.1 °C in Loch Etive. Whether these temperature differences among the three Scottish sites have any effect on the frequency of proximal + interstitial chiasmata, is a question that needs to be answered in a much deeper analysis, with a much larger sample of individuals and a more insightful kind of experiment.
